# Retinal Vascular Oxygen Saturation and Its Variation With Refractive Error and Axial Length

**DOI:** 10.1167/tvst.8.4.22

**Published:** 2019-08-07

**Authors:** Laurence Shen Lim, Xian Hui Lim, Licia Tan

**Affiliations:** 1 Singapore National Eye Centre, 11 Third Hospital Ave, Singapore 168751, Singapore; 2 Singapore Eye Research Institute, 11 Third Hospital Ave, Singapore 168751, Singapore

**Keywords:** myopia, imaging, retinal vessels, oxygenation, oximetry

## Abstract

**Purpose:**

To evaluate the relationships between refractive error, axial length (AL), and retinal vascular oxygen saturation in an adult population.

**Methods:**

This was a hospital-based, prospective observational study. The left eyes of phakic adult subjects without media opacity were analyzed. Subjective undilated manifest refraction was performed, and refractive errors were defined as myopia (spherical equivalent [SE], <−1 D), emmetropia (SE between −1 D and +1 D) and hyperopia (SE >+1 D). Retinal oximetry was performed using the Oxymap system (Oxymap Inc., Reykjavik, Iceland). Multivariate linear regression models were constructed to assess the relationship between retinal vascular oxygen saturation, SE, and AL obtained with optical biometry, with adjustments for age, sex, race, blood pressure, hyperlipidemia, and diabetes mellitus.

**Results:**

There were 85 subjects, with mean age of 66.1 ± 11.3 years. The majority were female (60%) and Chinese (84%). A total of 60% were myopic, 28% emmetropic, and 12% hyperopic. Mean SE was −5.29 ± 6.51 D and mean AL was 25.30 ± 2.99 mm. In multivariate analyses, more myopic SE and longer AL were associated with lower retinal arteriolar oxygen saturation (regression coefficient B = 0.61 [95% confidence interval, 0.28, 0.95], *P* = 0.001; and B = −1.13 [95% confidence interval, −1.71, −0.56], *P* < 0.001, respectively). Subjects with myopic SE and AL also had lower retinal arteriolar oxygen saturation than emmetropes and hyperopes (*P* = 0.03 and *P* = 0.02, respectively).

**Conclusions:**

Eyes with more myopic SE and longer AL have lower retinal arteriolar oxygen saturation.

**Translational Relevance:**

This study provides direct evidence of a link between retinal oxygenation and hypoxia and myopia by using a novel device that quantifies retinal vascular oxygenation in vivo.

## Introduction

The high prevalence of myopia in Asian adults is well established.^1^,^2^ The prevalence rate of myopia in Singaporean Chinese in the Tanjong Pagar Eye Study^3^ is amongst the highest in the world at 32.7%, with a large proportion (approximately 10%) with high myopia. High myopia is a major cause of legal blindness in many countries.^4^ Individuals with high myopia are at risk of developing various blinding complications.^5^,^6^ As such, myopia poses a significant public health concern.

Several studies have shown a variety of hemodynamic changes in the retinal vasculature in myopic eyes, and these changes have been postulated to contribute to the development of the complications of high myopia. Myopic eyes have been demonstrated to have attenuation of retinal vascular caliber.^7^–^9^ Shimada et al.^10^ have shown that this narrowing of blood vessel diameter in myopic eyes is associated with a decrease in retinal blood flow. This narrower retinal vascular caliber was also shown to be associated with elongated axial length (AL).^11^ Karczewicz et al.^12^ have further shown that the rate of retinal blood flow decreases with increasing refractive error in high myopia. Most studies have used retinal vessel caliber or retinal blood flow as surrogate markers of retinal tissue oxygen delivery and use, as direct assessments of retinal vascular oxygen content have been difficult.

Oxymap (Oxymap Inc., Reykjavik, Iceland) is an automated oximeter that is able to reliably assess the oxygen saturation in retinal vessel segments in vivo.^13^–^16^ Data on retinal vessel oxygen saturation in myopic patients are limited with inconsistent associations reported to date. Zheng et al.^17^ showed lower retinal arteriolar saturation in myopic eyes compared to normal eyes. Lower retinal arteriolar-venular difference (AVD) in eyes with high myopia and longer AL has been reported by Man et al.^12^ and Zheng et al.^17^ However, Liu et al.^18^,^19^ demonstrated higher arteriolar oxygen saturation levels with increasingly myopic refractive error in children less than 18 years old, whereas Yang et al.^20^ did not find correlations between arteriolar and venular oxygen saturation and myopia. These studies have found inconsistent relationships, and an important cause may be that the effect of ocular magnification on oxygen saturation measurement has not been addressed in these prior studies.^21^

The aim of this study was to evaluate the relationships between refractive error, AL, and retinal vascular oxygen saturation in an adult population.

## Methods

This was a hospital-based prospective observational study. Adult phakic patients aged at least 21 years old without media opacity as determined by a single clinical examiner were recruited from general outpatient clinics in the Singapore National Eye Centre.

All patients underwent slit lamp examination, refraction, biometry, and imaging with the Oxymap retinal oximeter. Interviewer-administered questionnaires were used to collect relevant sociodemographic data and medical history, including country and state of birth, marital status, education, occupation and current housing status, participants' lifestyle factors, history of smoking, eye symptoms, use of spectacles, current medications, systemic medical and surgical history, and family history of eye diseases.

Subjective undilated manifest refraction was performed by the same study optometrist to obtain a spherical equivalent (SE) refraction. SE was defined as the spherical power plus half the cylindrical power (negative cylinder).

AL was measured using optical biometry (IOLMaster version 3.01, Carl Zeiss; Meditec AG, Jena, Germany). The intraocular pressure was measured with noncontact airpuff tonometry. Blood pressure and heart rate were recorded using an automated sphygmomanometer (Dinamap model Pro Series DP110X-RW, 100V2; GE Medical Systems Information Technologies Inc., Westborough, MA) after the patient had been resting in a seated position for 5 minutes. Two readings were taken 5 minutes apart, with a third reading taken if the two differed by ≥10 mmHg systolic or ≥5 mm Hg diastolic.

Refractive categories were then defined as follows: emmetropia or hyperopia (SE, >−1 D), low myopia (SE, −1 D to −6 D), and high myopia (SE, <−6 D). Refractive categories were also defined according to the AL. We defined hyperopic eyes as AL ≤23 mm, emmetropic eyes as 23 mm > AL ≤ 25 mm, and myopic eyes as AL >25 mm.^22^

All study procedures were performed in accordance with the tenets of the Declaration of Helsinki as revised in 1989. Written informed consent was obtained from the subjects, and the study was approved by the Institutional Review Board of the Singapore Eye Research Institute.

### Retinal Oximetry

All patients underwent dilated fundus imaging to quantify retinal vascular oxygen saturation with the Oxymap system (Oxymap Inc.). The Oxymap system measures retinal vasculature oxygen saturation in the dark with a spectrophotometric retinal oximeter. First, a trained technician performed fundus photography in a darkened room. The dual wavelength, noninvasive Oxymap device consists of an optical adapter coupled to two digital cameras (1600 × 1200 square pixels). Fundus images captured were centered on the optic disc (OD) with 50° fields of view and taken with two different wavelengths of light—the oxygen insensitive wavelength of 586 nm and the oxygen sensitive wavelength of 605 nm. As light absorbance changes with the oxygen saturation, the optical density ratios correlate linearly with oxygen saturation. The proprietary device software (Oxymap version 3.1.4; Oxymap Inc.) processes the two images taken with the different wavelengths to generate a color-coded map. A single trained technician obtained all fundus images.

**Figure 1:**
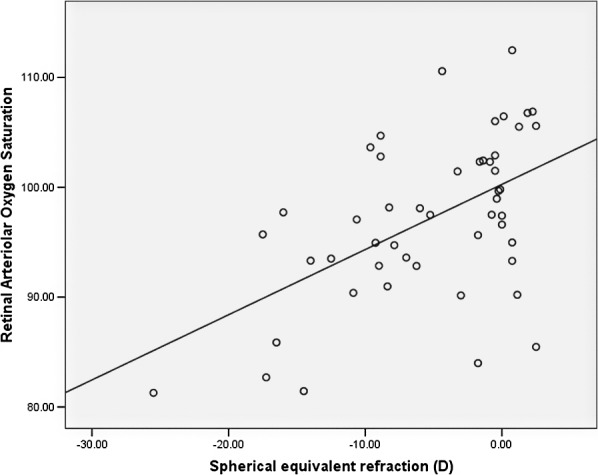
Scatterplot of retinal arteriolar oxygen saturation versus SE refraction.

A standardized protocol was followed for image analysis. Retinal arterioles and venules that fell within the measurement zone from 20 to 220 pixels from the OD margin were analyzed. The area within less than 20 pixels from the OD was not included in the measurement zone to avoid areas of peripapillary atrophy. The oxygen saturation in the retinal arterioles and venules with vessel width of more than 6 pixels and a minimum length of 100 pixels was measured. For branching vessels, the parent branch was measured. Daughter branches were also measured if the parent branch was less than 100 pixels in length. At vessel crossings, the distal segment was measured if it was more than 100 pixels in length. If the distal segment measured less than 100 pixels in length, the proximal segment was measured. The location of each vessel end point determined the quadrant localization assigned to each vessel. The overall quadrant retinal oxygen saturation level was calculated as the total retinal oxygen saturation in that quadrant, multiplied by the diameter of each vessel in the fourth power, and divided by the total sum of vessel diameters in the fourth power (Fig. 1). [corrected retinal vessel caliber = uncorrected retinal vascular caliber × (1 − 0.017 × SE refraction)].^23^ The difference between retinal arteriolar oxygen saturation and retinal venular oxygen saturation was calculated to give the AVD.

\newcommand{\bialpha}{\boldsymbol{\alpha}}\newcommand{\bibeta}{\boldsymbol{\beta}}\newcommand{\bigamma}{\boldsymbol{\gamma}}\newcommand{\bidelta}{\boldsymbol{\delta}}\newcommand{\bivarepsilon}{\boldsymbol{\varepsilon}}\newcommand{\bizeta}{\boldsymbol{\zeta}}\newcommand{\bieta}{\boldsymbol{\eta}}\newcommand{\bitheta}{\boldsymbol{\theta}}\newcommand{\biiota}{\boldsymbol{\iota}}\newcommand{\bikappa}{\boldsymbol{\kappa}}\newcommand{\bilambda}{\boldsymbol{\lambda}}\newcommand{\bimu}{\boldsymbol{\mu}}\newcommand{\binu}{\boldsymbol{\nu}}\newcommand{\bixi}{\boldsymbol{\xi}}\newcommand{\biomicron}{\boldsymbol{\micron}}\newcommand{\bipi}{\boldsymbol{\pi}}\newcommand{\birho}{\boldsymbol{\rho}}\newcommand{\bisigma}{\boldsymbol{\sigma}}\newcommand{\bitau}{\boldsymbol{\tau}}\newcommand{\biupsilon}{\boldsymbol{\upsilon}}\newcommand{\biphi}{\boldsymbol{\phi}}\newcommand{\bichi}{\boldsymbol{\chi}}\newcommand{\bipsi}{\boldsymbol{\psi}}\newcommand{\biomega}{\boldsymbol{\omega}}\begin{equation}\tag{1}{\rm{Mean\ oxygen\ saturation}} = {{{S_1} * d\kern 2pt_1^4 + {S_2} * d\kern 2pt_2^4 + {S_3} * d\kern 2pt_3^4 + {S_4} * d\kern 2pt_4^4 + {S_5} * d\kern 2pt_5^4 + {S_6} * d\kern 2pt_6^4 + {S_7} * d\kern 2pt_7^4 + {S_8} * d\kern 2pt_8^4} \over {d\kern 2pt_1^4 + d\kern 2pt_2^4 + d\kern 2pt_3^4 + d\kern 2pt_4^4 + d\kern 2pt_5^4 + d\kern 2pt_6^4 + d\kern 2pt_7^4 + d\kern 2pt_8^4}},\end{equation}

where *S_n_* = saturation of *n* vessel segment, and *d* = diameter of *n* vessel segment.

### Statistical Analysis

Multivariate linear regression models were constructed to assess the relationships between retinal arteriolar and venular oxygen saturation and AVD, and SE and AL, with adjustments for age, sex, race, blood pressure, hyperlipidemia, and diabetes mellitus. We regarded *P* values of less than 0.05 from two-sided tests as statistically significant. All statistical analyses were performed using SPSS version 16.0 (SPSS, Inc., Chicago, IL).

## Results

There were 85 patients in total. The majority were female (60%) and Chinese (84%). The left eye of each subject was analyzed. The mean age of the subjects was 66.1 ± 11.3 years.

The mean SE was −5.29 ± 6.51 D, and the mean AL was 25.30 ± 2.99 mm. SE and AL were strongly correlated (Pearson correlation coefficient *r* = −0.88, *P* < 0.001). The majority were myopic (60%), 28% were emmetropic, and 12% were hyperopic. The demographic characteristics of the population are summarized in Table 1.

**Table 1:** Demographic Characteristics of Study Population

Parameter	Value
Age (years), mean ± standard deviation	66.1 ± 11.3
AL (mm), mean ± standard deviation	25.30 ± 2.99
SE (D), mean ± standard deviation	−5.29 ± 6.51
Sex, no. (%)	
Male	34 (40)
Female	51 (60)
Race, no. (%)	
Chinese	71 (84)
Other races	14 (16)
Refractive error status (by dioptric power), no. (%)	
Hyperopic or emmetropic	10 (12)
Low myopic	24 (28)
High myopic	51 (60)
Refractive error status (by AL), no. (%)	
Hyperopic	18 (21)
Emmetropic	32 (38)
Myopic	35 (41)

In multivariate analyses adjusting for age, sex, race, mean arterial blood pressure, and presence of diabetes mellitus and dyslipidemia, more myopic SE was associated with lower retinal arteriolar oxygen saturation (regression coefficient B = 0.61 [95% confidence interval (CI), 0.28, 0.95], *P* = 0.001) (Table 2). Scatterplots of SE versus retinal arteriolar oxygen saturation are shown in Figure 1.

**Table 2:** Multivariate Analysis of the Association Between SE Refraction and Retinal Arteriolar Oxygen Saturation

Covariates	Regression Coefficient (B)	95 % CI for B	*P* Value
Age (years)	-0.28	−0.21, −0.16	0.76
Sex	−1.26	−5.73, 3.21	0.58
Race	0.83	−3.07, 4.73	0.67
Diabetes mellitus	−1.62	−6.52, 3.28	0.51
Dyslipidemia	−0.30	−5.36, 4.77	0.91
MABP (mm Hg)	−0.003	−0.17, 0.16	0.97
SE (D)	0.61	0.28, 0.95	0.001

MABP, mean arterial blood pressure.

**Figure 2:**
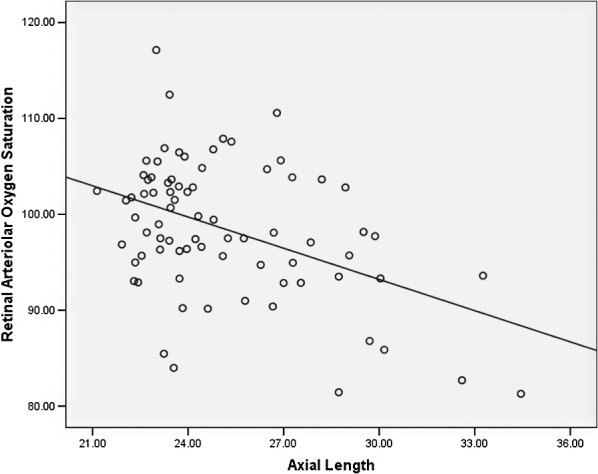
Scatterplot of retinal arteriolar oxygen saturation versus AL.

Longer AL was also associated with lower retinal arteriolar oxygen saturation in multivariate analysis adjusting for the above-mentioned factors (B = −1.13 [95% CI, −1.71, −0.56], *P* < 0.001) (Table 3). Scatterplots of AL versus retinal arteriolar oxygen saturation are shown in Figure 2.

**Table 3:** Multivariate Analysis of the Association Between AL and Retinal Arteriolar Oxygen Saturation

Covariates	Regression Coefficient (B)	95% CI for B	*P* Value
Age (years)	0.31	−0.11, 0.17	0.67
Sex	−2.75	−5.84, 0.33	0.08
Race	1.77	−0.84, 4.38	0.18
Diabetes mellitus	−1.49	−4.95, 1.98	0.40
Dyslipidemia	−1.76	−5.38, 1.85	0.33
MABP (mm Hg)	−0.22	−0.13, 0.09	0.69
AL (mm)	−1.13	−1.71, −0.56	<0.001

**Figure 3:**
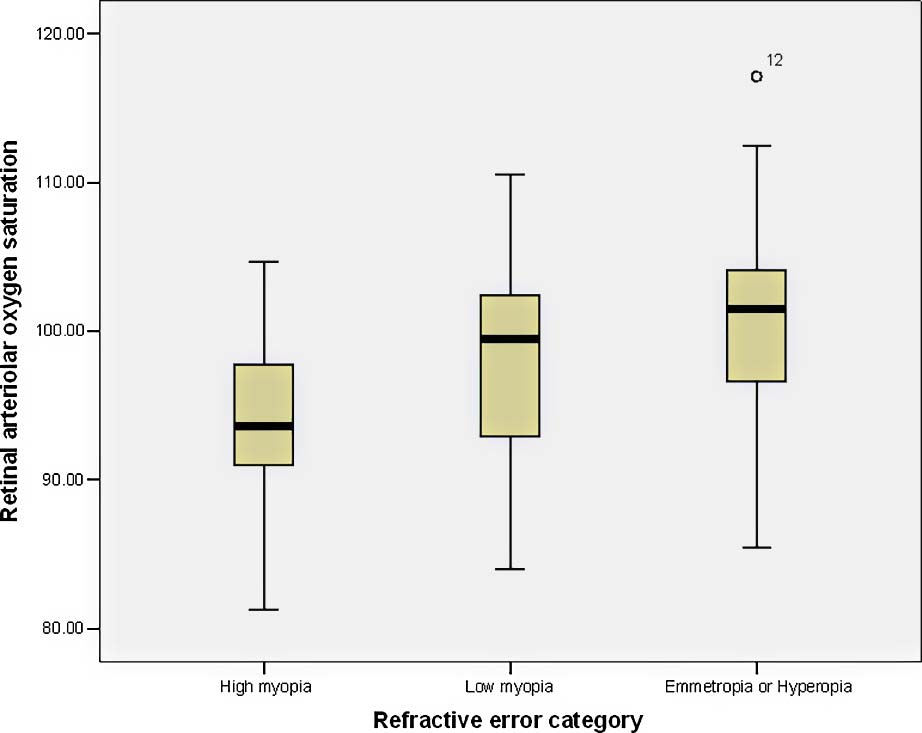
Boxplots of retinal arteriolar oxygen saturation by refractive error category.

Subjects with highly myopic SE had lower retinal arteriolar oxygen saturation, compared to low myopic and emmetropic and hyperopic subjects (*P* < 0.001). In particular, there was a significant difference between the groups with myopic SE and emmetropic SE (mean difference, −6.90 [95% CI, −11.01, −2.78]; *P* < 0.001). There were no significant differences between the groups with high myopia and low myopia, or low myopia and emmetropia or hyperopia (Fig. 3).

**Figure 4:**
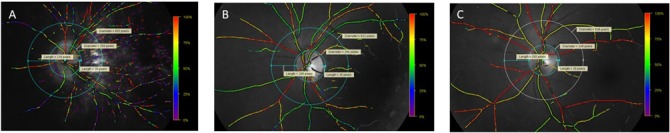
Fundus images for (A) highly myopic eye, (B) low myopic eye, and (C) hyperopic eye. The retinal arteriolar saturation was 85.8% in the highly myopic eye, 102.3% in the low myopic eye, and 106.9% in the hyperopic eye.

Representative fundus images from a highly myopic, low myopic, and a hyperopic eye respectively are shown in Figure 4.

When grouped by AL, subjects with myopic AL also had a lower retinal arteriolar oxygen saturation (*P* = 0.04).

There were no associations between SE or AL and venular oxygen saturation or AVD.

## Discussion

Our study measured and compared the retinal vasculature oxygen saturation in patients with varying refractive errors ranging from hyperopia to high myopia. In this study, we have shown that eyes with more myopic refractive errors and longer ALs have lower retinal arteriolar oxygen saturation. Venular oxygen saturation and AVD were not associated with refractive error or AL changes.

A few studies have investigated the oxygen saturation of retinal vessels in myopes with the Oxymap retinal oximeter, with inconsistent results. A cross sectional study by Zheng et al.^17^ comparing high myopes with emmetropes found that eyes with high myopia had significantly lower retinal arteriole oxygen saturation. In further subgroup analyses, patients with worse myopic maculopathy (grade ≥M2) were noted to have lower retinal arteriolar saturation than emmetropes, whereas eyes with mild myopic maculopathy (grade <M2) did not. Chen et al.^24^ reviewed 17 high myopes with mean SE of −14.2 ± 4.49 D pre- and postimplantable collamer lens surgery. In this study, the baseline retinal arteriolar oxygen saturation level in the highly myopic patients was 93.8% ± 4.6%, which was lower than the normal population measured by Geirsdottir.^13^ However, in another cross-sectional study aiming to provide normative data for retinal vessel oxygen saturation levels in 122 young Chinese subjects aged 5 to 18 years with mean refractive error of −3.25 ± 2.49 D (range, −8.88 to +3.13),^18^ higher retinal arteriolar oxygen saturation levels correlated with more myopic SE. Liu et al.^25^ reported the normative retinal oxygen saturation values in 1461 Chinese children aged 7 to 19 years and found that longer AL was associated with higher arteriolar saturation and AVD. A cross-sectional study by Yang et al.^20^ included subjects aged 19 to 30 and found no significant difference in retinal blood saturation between the group with refractive error less than −3.00 D and the other with refractive error of −3.00 D and above. However, multivariate analysis showed a negative correlation between arteriolar and venular oxygen saturation, with the product of diopter and oxygen perfusion pressure, and a positive correlation between arteriolar oxygen saturation and the product of diopter and vessel diameter.^20^

These studies had inconsistent findings regarding the association between retinal arteriolar oxygen saturation and myopia. Our findings of lower retinal arteriolar oxygen saturation in myopia were similar to Zheng et al.^17^ and Chen et al.^24^ The studies by Liu et al.^18^,^25^ in younger populations, however, both found contrasting trends of higher arteriolar saturation with more myopic refraction and longer AL. One of the key factors affecting the relationship, thus, appears to be age, with studies in adult populations, including our current study, finding lower arteriolar saturation with myopia, and studies in children finding the opposite. This has been attributed to the development of atrophy of the retina and choriocapillaris with time in adult myopic eyes, leading to reduced oxygen requirements. A second important factor is that of the effect of refractive error on ocular magnification. This factor, although cited as possibly affecting their results, has largely been unaccounted for in the studies by Liu and Zheng. It is likely to be an important factor, as it has been reported that vessel oxygen saturation is correlated negatively with vessel diameter.^21^ Yang et al.^20^ noted interactions between refractive error and vessel diameter, and this was postulated to affect oxygen retinal saturations. As such, they include the product of the dioptric power of the eye and vessel diameter as an “interaction variable” in their analyses but were unable to conclusively determine any relationship other than a negative correlation between of retinal vasculature oxygen saturation levels and the product of the diopter and vessel diameter. Our study has attempted to address the issue of ocular magnification directly by adjusting for the effect of ocular magnification on retinal vascular caliber measurement by using the Bengtsson formula prior to calculating the mean oxygen saturation in the vessels.

We did not find any significant decrease in venular oxygen saturation levels in myopia, Our findings are consistent with those of Zheng et al.^17^ who postulated that any expected reduction in venular oxygen saturations from decreasing blood flow in myopic retinal venules may be compensated for by an increase in countercurrent exchange of oxygen in the optic nerve. On the contrary, Liu et al.^18^ found increasing retinal venular saturations in myopia. Again, the younger age, lower refractive range of their population and ocular magnification effects could account for this difference in findings, especially because they also demonstrated that venular oxygen saturation levels increased with age. Our findings of no association between AVD and myopia were similar to Liu et al. Other studies have had differing findings. Man et al.^26^ conducted a cross-sectional study that concluded that eyes with increased AL had decreased AVD and corresponding reductions in retinal function based on multifocal electroretinogram P1 amplitude. In this study, a significant decrease in AVD was seen in longer eyes. Zheng et al.^17^ also found lower AVD in myopic eyes than in emmetropic eyes. It was proposed that the decreased AVD reflected a lower level of oxygen consumption due to lower oxygen demand from fewer functional healthy neurons in myopia. In both these studies, the subjects were young adults (mean age, ∼30 years), unlike the much older population in our study (mean age, 66 years), and our study included a predominantly Asian population that may account for the differences in findings from these other studies.

This study included a relatively large cohort, with clearly defined protocols to limit examiner bias. All fundus photographs were obtained by a single trained technician, and analysis of all images were performed by two assessors who followed the standardized image analysis protocol. Both assessors were also blinded to refractive error or AL of the eyes imaged, and only eyes with clear media were included to allow for high-quality image capturing. The effects of ocular magnification on vessel diameters, which is critical in any analysis involving refractive error, were also adjusted for using the well-described Bengtsson formula. Limitations of our study include its cross-sectional design, which prevents conclusions on the causality and temporal relationship between myopia and lower retinal arteriole oxygen saturation. There was also the possibility of selection bias in a clinic-based setting. Our population was also exclusively of Asian ethnicity, and ethnic variations in fundal pigmentation could have affected the measurements. These biases are, however, nondifferential and should not have affected the associations found.

In conclusion, lower retinal arteriolar oxygen saturation is associated with myopic refractive errors. Our results suggest a role for hemodynamic changes in the retinal circulation in the pathogenesis of myopia. Further longitudinal study is warranted to determine the temporal relationship between retinal oxygenation and the development of myopia and its complications.
